# Precautionary Measures: Performing ERCP on a Patient With Juxtapapillary Duodenal Diverticula (JPDD)-Related Biliary Stone After COVID-19 Lockdown Restriction Lifted in Wuhan, China

**DOI:** 10.3389/fmed.2020.00564

**Published:** 2020-09-04

**Authors:** Qian Chen, Min Yang, Guang-quan Liao, Yu-er Zhao, Dao-yuan Yue, Yue Yuan, Bin Cheng, Hua Qin

**Affiliations:** ^1^The Division of Gastroenterology and Hepatology, Department of Internal Medicine at Tongji Hospital, Tongji Medical College, Huazhong University of Science and Technology (HUST), Wuhan, China; ^2^The Department of Laboratory Services at Tongji Hospital, Tongji Medical College, Huazhong University of Science and Technology, Wuhan, China; ^3^Tongji Medical College, Huazhong University of Science and Technology, Wuhan, China

**Keywords:** ERCP (endoscopic retrograde cholangiopancreatography), COVID-19, post-coronavirus outbreak, personal protection equipment (PPE), Healthcare workers (HCW)

## Abstract

On April 8, 2020, after nearly 3 months of battling against the outbreak of COVID-19, Wuhan, where the pandemic began, began easing lockdown restrictions. However, given that asymptomatic carriers could continue to lead to transmission of COVID-19 during the very early stages, the endoscopists have taken precautions and conduct risk assessments to perform endoscopic intervention in this transition stage. Here, we have reported an urgent ERCP in a patient with acute pancreatitis secondary to JPDD-related biliary stone. Based on our experiences, the objective is to provide practical suggestions for the safe resumption of ERCP procedures in the setting of the COVID-19 pandemic with specific focus on patient risk assessment, personal protection equipment (PPE), and dress code modalities, all of which have been implemented in our hospital to reduce the risk of viral transmission.

## Background

The new strain of coronavirus, SARS-CoV-2, was first extracted in December 2019 from the lower respiratory tract samples of several pneumonia patients in our city, Wuhan, Hubei province, China (1–3). On March 11, 2020, the World Health Organization (WHO) declared the infection of SARS-CoV-2 with the official name COVID-19 (novel coronavirus disease-2019) a pandemic, highlighting the significance of its worldwide spread. The classic description of COVID-19 is as a respiratory illness that manifests with fever, dry cough, and dyspnea on exertion. However, fecal–oral transmission may be part of the COVID-19 clinical picture (4, 5). Accordingly, the endoscopy departments face a significant risk of diffusion of respiratory diseases that can be spread *via* an airborne route, including aspiration of oral and fecal material via endoscopes. Healthcare workers (HCW) have a high risk of infection; the infected HCW in Wuhan city consisted of 29% of COVID-19 patients at the beginning (6). Since then, the use of personal protective equipment (PPE), such as gloves, mask, goggles, face shields, gowns, and hairnets, are strongly advocated among all medical societies for conducting physical examination and clinical procedures (7).

The gastroenterologists and the HCW in endoscopic fields have remarkable risk to be exposed to either respiratory or gastrointestinal fluids from patients during the endoscopy procedures (7, 8). To minimize human-to-human transmission and to best protect HCW, our hospital, which used to be a part of the coronavirus epicenter, has cut its ambulatory endoscopy practice and developed a screening system that only allows urgent endoscopies being performed during the COVID-19 outbreak. After lifting lockdown restrictions, the endoscopy services have resumed, and the hospitals and healthcare facilities take precautions for endoscopy procedures amid concerns over asymptomatic carriers who could potentially lead to second COVID-19 outbreak.

Diverticula located near the major duodenal papilla are termed juxtapapillary duodenal diverticula (JPDD) (9). Although JPDD is common and rarely give rise to severe complications, it tends to act as an independent risk factor for biliary stone formation. Furthermore, JPDD plays an etiological role in the development of acute pancreatitis, and the underlying pathophysiological mechanisms include biliary stone-induced obstruction, pressure changes in the sphincter of Oddi, and obstruction of pancreatic outflow directly caused by extraluminal diverticula compression, respectively (10). For patients with predicted severe acute biliary pancreatitis, whether or not cholangitis is present, urgent therapeutic endoscopic retrograde cholangiopancreatography (ERCP) within 72 h of admission has been recommended by several guidelines, as fewer complications tend to develop (11–14). Here we share our experience of conducting an urgent ERCP on a 73-year-old patient who developed acute pancreatitis secondary to JPDD-related biliary stone. Our experience may provide practical suggestions to minimize the transmission of COVID-19 during an ERCP procedure.

## Case Presentation

**Figure 1:**
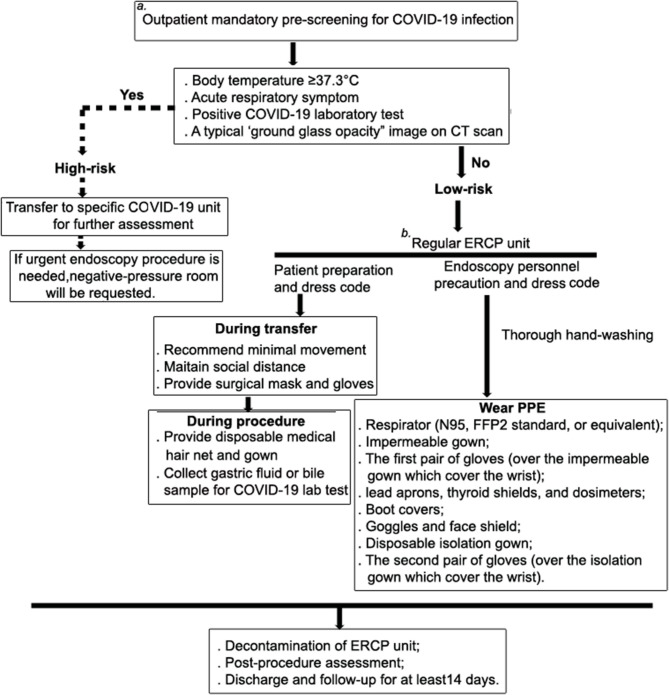
Patient management and risk assessment and detailed workflow during ERCP procedure. To minimize human-to-human transmission and to best protect HCW, our hospital, which used to be the part of the coronavirus epicenter, has cut its ambulatory endoscopy practice and developed a screening system to only allow urgent endoscopy being performed during the COVID-19 outbreak. **(a)** Pre-assessment for COVID-19 is mandatory and conducted in the out-patient department, and it includes inquiries into potential contact history (whether contacted with a suspected or laboratory-confirmed COVID-19 patient in the last 2 weeks), a patient symptom check (body temperature ≥37.3°C, coughing or shortness of breath and/or other symptoms of acute respiratory symptom are highly suspected), laboratory test (a nasopharyngeal swab specimen for COVID-19 RNA test and serological tests for COVID-19 antibody), and a chest computed tomography (CT) scan (a typical “ground glass opacity” image is highly suspected). If the patient meets one of these criteria, they will be categorized as being at high risk of infection, and they will be administered at a separated unit for all patients with respiratory symptoms. The negative-pressure room will be requested for undergoing therapeutic procedure. **(b)** At the ERCP unit, patients are further assessed for suitability for conscious sedation. Those who have cardio-pulmonary disease, difficult airway, morbid obesity, and significant GERD are not eligible for conscious sedation.

A 73-year-old female presented at the outpatient department with a 2-day history of upper abdominal pain after a meal. She has no pre-existing conditions or major past medical history. Before admission, she went through the mandatory pre-screening assessment (Figure 1), which has been implemented at our hospital through the COVID-19 outbreak, including inquiry of potential contact history (whether contacted with a suspected or laboratory-confirmed COVID-19 patient in the last 2 weeks); patient's symptom check (body temperature ≥37.3°C, coughing or shortness of breath and/or other symptoms of acute respiratory symptom are highly suspected); laboratory test (a nasopharyngeal swab specimen for COVID-19 RNA test and serological tests for COVID-19 antibody) (6); and a chest computed tomography (CT) scan (a typical “ground glass opacity” image is highly suspected), respectively.

**Figure 2:**
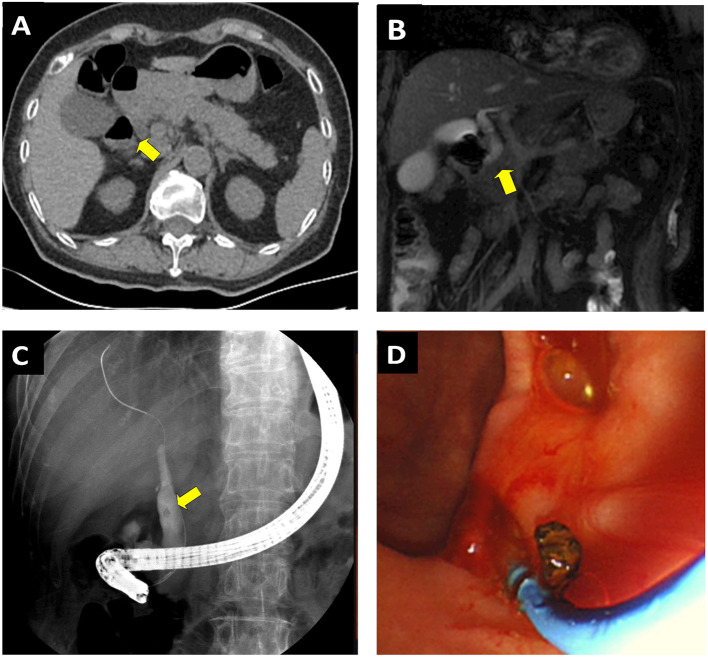
Performing ERCP on a 73-year-old female patient presented with acute pancreatitis secondary to JPDD related CBD stone. **(A)** CT scan shows the inflammation and swelling of the pancreas and a mildly enlarged gallbladder. A diverticular pouch was present at the junction of second and third portions of the duodenum (yellow arrows). **(B)** MRCP coronal haste thin slice image confirmed shows the presence of periampullary diverticulum, which causes extrinsic compression upon the CBD (yellow arrow). Note the possible CBD stone associated with dilation of the distal end of CBD. **(C)** ERCP confirm the CBD stone and divericulum exerting compression upon the CBD outlet. **(D)** The stone was successfully removed though the ERCP procedure.

The patient was categorized as having a “low risk” of COVID-19 infection and was subsequently admitted to the GI unit. During routine physical examination, her vital signs were stable, whereas moderate rebound tenderness appeared at the upper abdominal region. The blood chemistry panel showed prominently elevated amylase (5,082 IU/L) and lipase (>3,000 IU/L) levels, suggesting pancreatitis. Bilirubin level and the lipid profile were normal, with the mild increase of gamma-glutamyl transpeptidase (GGT, 59 U/L), alamine amino transferase (ALT, 95U/L), and aspartate amino transferase (AST, 95U/L), respectively. In addition, complete blood count showed severe inflammation with increased white blood cell (20.5 × 10^9^/L) and neutrophil (19.1 × 10^9^/L) counts. An abdominal CT scan indicated inflammation and swelling of the pancreas, a mildly enlarged gallbladder, as well as a slightly dilated common biliary duct (CBD) (Figure 2A). Notably a diverticular pouch was present at the junction of second and third with no obvious stone identified at that time portions of duodenum. To further rule out the possible biliary or extrabiliary obstructive pathology, the patient was referred to MR cholangiopancreaticography (MRCP) examination. MRCP coronal haste thin slice image confirmed the presence of duodenal diverticular partially compressing the distal end of CBD and resulting in dilation of its proximal part (Figure 2B). The maximal diameter of the diverticula was 2.67 cm. An ERCP may be sufficient to identify the presence of small stones (and subsequently remove them) or, alternatively, to place a stent inside the duct to restore bile flow.

## Patient Preparation and Dress Code

The patient was informed of management options and agreed to endoscopic interventions. She was also acknowledged to have potential exposure risks to COVID-19 in the hospital environment. Surgical mask and gloves were provided to the patient and the relative who was responsible for transfer her to the ERCP unit. In addition, the patient was also provided with a disposable medical hair net and gown. They were further advised to minimize movement while waiting for the procedure to minimize the risk of contamination.

Prior to the ERCP procedure, the patient's status of COVID-19 was verified among the ERCP team. To limit the exposure risk for HCW, general anesthesia with tracheal intubation or deep sedation, which normally requires the anesthesia personnel to stay in the procedure room, was not applied in the current setting. Pre-ERCP screening was therefore critical to assess the patient's suitability to undergo conscious sedation. With regards to this, the patient had no cardio-pulmonary disease, no difficulties related to the airway, no morbid obesity, and, furthermore, no significant gastroesophageal reflux disease (GERD), which will probably cause an increased risk of developing complications during and after the ERCP procedure. Ten minutes before the procedure, intravenous administration of diazepam (5 mg) and dezocine (2.5 mg) was used to generate effects of anesthesia for the patient. In addition, antispasmodic (phloroglucinol, 20 mg) was given to reduce duodenal motility for the procedure. The vital parameters [heart rate (HR), blood pressure (BP), and respiration rate, (SpO2)] were monitored throughout the procedure.

## Endoscopy Personnel Precautions and Dress Codes During an ERCP Procedure

Although performing endoscopic procedures in a negative-pressure room during the COVID-19 outbreak was recommended among several gastroenterological endoscopy societies (7, 15, 16), this is not available in most endoscopy facilities around the world. We equipped an operative room with a negative-pressure system in a separate unit to be used for all patients with respiratory symptoms. Given that the current patient was categorized as being at a low risk of infection, she was not transferred to the negative-pressure room.

**Figure 3:**
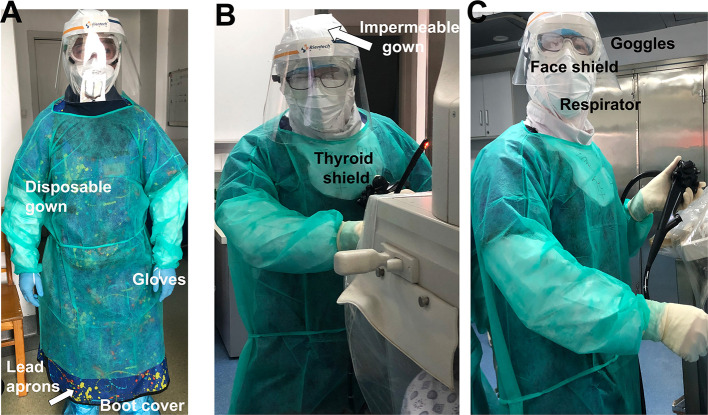
**(A–C)** HCWs adhere to Level 2 biosafety requirement during the procedure. ERCP Endoscopy personnel precautions and dress code as follows: prior to ERCP procedure, the patient's status of COVID-19 was verified among the ERCP team. HCW wore PPE in the following order: respirator (N95, NK95, or the equivalent); impermeable gown; a first pair of gloves (over the impermeable gown that cover the wrist); lead aprons, thyroid shields, and dosimeters; boot covers; goggles and face shield; disposable isolation gown; and a second pair of gloves (over the isolation gown which cover the wrist). Washing hands with soap and water or alcohol-based hand rub were mandatory before and after patient interaction, contact with potentially infectious sources, and before putting on and upon removal of PPE.

All HCW at the hospital have received appropriate training on hand hygiene and use of PPE prior to procedures (7, 8). The endoscopists and assistance wore PPE by reviewing the Asian Pacific Society for Digestive Endoscopy (APSDE) guidelines, American Society for Gastrointestinal Endoscopy (ASGE) guidelines, and CDC recommendations for ERCP (8, 15–17). Washing hands with soap and water or alcohol-based hand rub were mandatory before and after patient interaction, contact with potentially infectious sources, and before putting on and removing PPE. The step-by-step approach for wearing PPE is as follows (Figures 3A–C): wear a respirator [either N95, the US standards for respirator masks, or NK95, the Chinese standards for respirator masks, or the equivalent, which are rated to capture 95% of tiny particles (0.3 micron particles, to be exact)]; wear an impermeable gown; wear the first pair of gloves so they cover the impermeable gown, which cover the wrist; wear lead aprons, thyroid shields, and dosimeters; wear boot covers; wear goggles and a face shield; wear a disposable isolation gown; wear a second pair of gloves over the isolation gown so they cover the wrist.

At ERCP, JPDD was observed, and a duodenoscopy identified the papilla located on the edge of diverticular fundus (Figure 2C). ERCP was performed in the usual fashion with selective biliary cannulation and injection of contrast material into common bile. A small stone was visualized in the distal CBD where it was juxtaposed against the diverticulum. To remove the stone, the endoscopist performed a small sphincterotomy in conjunction with balloon dilation, and subsequent removal of the stone was achieved with balloon extraction (Figure 2D). A 6F endoscopic nasobiliary drainage (ENBD) tube was placed to drain remnant stone. After the procedure, the patient's respiratory and cardiac signs were carefully monitored in the GI ward. Physical distancing was emphasized; we permitted only one relative or guardian per patient, and they were given masks and a separate room to other patients. All the nurses and healthcare providers were tested for COVID-19 and had to show negative results prior to returning work; we had mandatory education and training on infection measures, including hand hygiene and use of PPE. A total of 2 days later, the patient's amylase and lipase levels returned to normal, and the EBD tube was thus withdrawn. In addition, a sample of bile collected during ERCP procedure was tested for COVID-19 serology; it showed negative results. The possible ERCP-related complications, including pancreatitis, infection of the bile ducts or gallbladder, hemorrhage, and perforation in the bile or pancreatic ducts, were not observed. The patient was discharged afterwards and followed up 2 weeks later to assess whether she developed any respiratory symptoms and to further assess her progress after the procedure. A total of 2 months later, she was further followed up at an outpatient department. There was no retaining stone detected in CBD by CT scan, and no evidence of post-ERCP pancreatitis or other ERCP-related complications developed during the follow-up.

## Discussion

Duodenal diverticula are bulging pouch-like herniations in the duodenal wall, and those located near the major duodenal papilla are termed JPDD. JPDD are acquired lesions, and their presence rises with increasing age. Although they are usually asymptomatic, the association with biliary or pancreatic disease is not uncommon, and this includes choledocholithiasis, perforation, acute/chronic pancreatitis, bleeding, CBD obstruction, and rarely carcinoma (9). In particular, JPDD plays a major role in biliary stone disease in which the bile de-conjugation by diverticula's compression may likely act as the initial step leading to the precipitation of calcium bilirubinate and formation of pigment stones (18, 19). The further mechanical obstruction and sphincter of Oddis dysfunction subsequentially increase the risk to develop acute pancreatitis. Preforming ERCP and endoscopic sphincterotomy are widely accepted as the first-line therapy to remove bile duct stones and explicitly benefit those with the etiology of acute pancreatitis, and accordingly, urgent ERCP within 72 h is required to reduce the risk of developing acute pancreatitis-associated complications (11–14). JPDD has been previously considered as a risk factor not only for cannulation difficulty during ERCP, but it is also linked to developing complications upon endoscopic sphincterotomy, bile duct stone retention, as well as recurrence after an ERCP procedure. However, recent studies highlight the technical capability and suggest experienced endoscopists can overcome the anatomical difficulties to safely and successfully conduct cannulation and endoscopic sphincterotomy; furthermore, there is no increased risk of hemorrhage following sphincterotomy in patients with periampullary diverticulum compared to those without one (20). During the COVID-19 pandemic, acute biliary obstruction requiring stenting and acute cholangitis are the only hepato-pancreaticobiliary disorders commonly recommended by many international or national endoscopy societies for demanding urgent ERCP (21), including the British Society of Gastroenterology (BSG) guidelines, APSDE guidelines, and ASGE guidelines (7, 15, 16). Here we presented the first case of acute pancreatitis secondary to JPDD related CBD stone at the post-outbreak stage of which COVID-19 is on the way to be fully controlled, whereas urgent ERCP was necessary to significantly improve the patient's outcomes. We hereby suggest the ERCP procedure for acute biliary pancreatitis should also be considered for urgent endoscopic intervention during the outbreak. Nevertheless, it is only conducted after risk stratification and careful pre-screening of the patient. Though the current case allows us to gain such experience, a lack of similar cases during the pandemic indeed prevents us from further assessing the additional challenges the HCW may face.

The current case was categorized as a low-risk COVID-19 patient. However, we adhered to Level 2 biosafety requirement when performing ERCP, and this is partly due to the aerosol-generating nature of the procedure. In addition, taking precautions to consider those asymptomatic carriers could lead to transmission of COVID-19 during very early stage (22), we emphasize the use of full PPE to protect our endoscopic personnel. We further highlight potential modifications as follows based on our experiences. First, before admission, the mandatory pre-screening assessment is implemented (which will last throughout the COVID-19 outbreak and after lockdown restriction are lifted). Second, the hospital has been reconstructed and divided into two sections, one for “low-risk” patients and the other for “high-risk” patients who will be administered at a separated section where all the patients with respiratory symptoms reside. Third, the patients with a “low risk” of COVID-19 are admitted to the GI unit prior to ERCP procedure. Furthermore, until now in Wuhan city, during the pandemic or in the post-COVID-19 period, pre-screening tests are required for both the patient and the patient's guardian or carer. The patient' relatives or guardian have to be at ‘low risk’ of COVID-19 to stay in the hospital. Fourth, HCW must adhere to Level 2 biosafety requirement during the procedure given the aerosol-generating nature of the virus and the precautions laid out for asymptomatic carriers. Fifth, to limit the exposure risk for HCW, general anesthesia with tracheal intubation or deep sedation, which normally requires the anesthesia personnel staying in the procedure room, are not currentl applied. Alternatively, conscious sedation is used after carefully pre-ERCP screening the patient's suitability.

We noted that one of the disadvantages related to usage of PPE was wearing goggles and a face shield together, as it can cause the lenses to fog up quickly. To prevent goggles from fogging, we used a small drop of a liquid soap to rub the lenses. Furthermore, the current design of face shields may not be able to cover the lower face region when endoscopists raise their heads (Figure 3C). This could be a potential risk for HCW while aerosolization appears. A modified design for the face shield could therefore be a solution.

Together, based on our practical experience and published guidelines, we strategically assigned HCW during an uncertain time to minimize concomitant exposure and applied the triage workflow throughout the urgent ERCP. Success in preventing COVID-19 transmission was achieved.

## Data Availability Statement

The raw data supporting the conclusions of this article will be made available by the authors, without undue reservation.

## Ethics Statement

Ethical review and approval was not required for the study on human participants in accordance with the local legislation and institutional requirements. The patients/participants provided their written informed consent to participate in this study. Written informed consent was obtained from the individual (s) for the publication of any potentially identifiable images or data included in this article.

## Author Contributions

GL and MY assisted endoscopic procedures in this study. YZ and YY collected and analyzed the clinical data. DY provided laboratory test and interpreted the results. HQ and BC supervised the study, performed the procedure and further gave valuable advice. The manuscript, images and their associated description were drafted by QC. All authors read and approved the manuscript.

## Conflict of Interest

The authors declare that the research was conducted in the absence of any commercial or financial relationships that could be construed as a potential conflict of interest.
